# Expanding Uranium Oxide Hydrate Frameworks toward Early Lanthanides: Cases for Pr(III) and Nd(III) Ions

**DOI:** 10.1021/acsomega.5c02821

**Published:** 2025-08-20

**Authors:** Maria K. Nicholas, Timothy A. Ablott, Jeremy Wykes, Brendan J. Kennedy, Yingjie Zhang

**Affiliations:** † School of Chemistry, The University of Sydney, Camperdown, NSW 2006, Australia; ‡ 5419 Australian Nuclear Science and Technology Organisation, Locked Bag 2001, Kirrawee DC, NSW 2232, Australia; § Australian Synchrotron, ANSTO, 800 Blackburn Road, Clayton, VIC 3168, Australia

## Abstract

We report the hydrothermal syntheses and structural and spectroscopic characterization of two new uranium oxide hydrate frameworks (UOHFs) with either Pr^3+^ or Nd^3+^ ions, Pr_1.5_(H_2_O)_6_[(UO_2_)_10_UO_13_(OH)_4_] (**UOHF-Pr**) or Nd_1.5_(H_2_O)_6_[(UO_2_)_10_UO_13_(OH)_4_] (**UOHF-Nd**). Both UOHFs crystallize in the orthorhombic *C*222_1_ space group and display needle crystal morphologies. Their crystal structures are composed of β-U_3_O_8_-type layers connected by double uranium polyhedra to form the frameworks, with disordered Pr^3+^/Nd^3+^ ions within the framework channels, as revealed by synchrotron single-crystal XRD. The presence of pentavalent uranium in both UOHFs was confirmed by a combination of diffuse reflectance and synchrotron X-ray absorption spectroscopies. The formation and stabilization of UOHF-Ln with earlier lanthanide ions in high symmetry structures are confirmed, and the strong correlation of the structural trends with the ionic radius of the lanthanide cation has been further validated. This work further emphasizes the complex uranium chemistry in the presence of lanthanide ions and has implications for uranium geochemistry and possible alterations of spent nuclear fuel under geological disposal.

## Graphical abstract

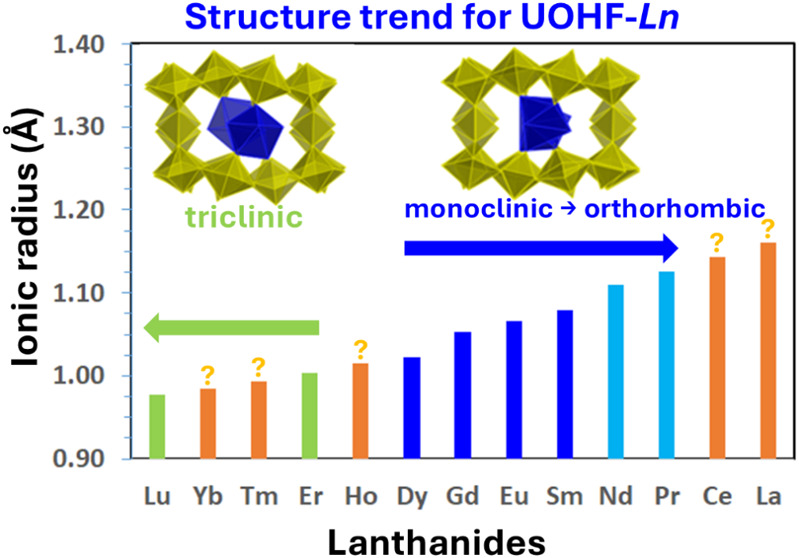

## 1 Introduction

Studying the alteration products of UO_2_ has been established as a means of better understanding the chemistry that spent nuclear fuel (SNF) stored in a geological repository undergoes when exposed to oxidation conditions, such as in an accident scenario.^1–3^ In nature, examining the oxidation and hydration of the mineral uraninite, a natural UO_2+x_ species, has revealed that uranium oxide hydrates (UOHs) are a dominant species in the early stage alteration pathway.^4,5^ The observation that such hydrated uranium phases have been found on the surface of SNF when subjected to oxidative conditions highlights the importance of studying UOH materials.^6^

The dominant uranium species in these UOH materials is U­(VI), present as the uranyl [UO_2_]^2+^ cation, which has two strongly coordinated uranyl oxygens in the axial positions. The uranyl cations can coordinate to neighboring uranium centers through their equatorial oxygens to form tetragonal, pentagonal, or hexagonal bipyramidal polyhedra, giving rise to layered (sheet) structures.^7–9^ These sheets can take on a variety of topologies, most often closely resembling α- and β-U_3_O_8_, but in some materials, they take on unique topologies.^10–12^ Importantly, such materials can take up secondary metal cations from the environment and incorporate these into the interlayer region, with layered structures containing alkali, alkaline earth, transition metals, p-block metals, and lanthanides all reported.^13–21^

A recent focus has been toward a subset of these materials, called uranium oxide hydrate frameworks (UOHFs), wherein the uranyl polyhedra layers are bridged by additional uranium polyhedra to form channels that extend in one dimension (1D) throughout the material. The secondary metal cation can be incorporated in these framework channels.^22^ The incorporation of alkaline earths, transition metals, and monovalent cations, such as ammonium, has been reported,^23–25^ but most dominant among these incorporated metal species are those of the lanthanides.^26–28^ Lanthanide (Ln) elements are often isolated in proximity to uranium in nature and, when considering an SNF system, are guaranteed to be in the immediate surroundings of UO_2_ if exposed to oxidation conditions.^5^ They are also an invaluable tool as surrogates to actinides such as Am^3+^ and Cm^3+^, highly radiotoxic minor actinides which are the subject of considerable effort in the field of radionuclide encapsulation and sequestration.^29,30^

Linking of the layers in UOHFs through the additional uranium polyhedra results in a marked increase in their stability compared to the corresponding layered structures, as evidenced by their stability under electron beam irradiation under high vacuum, such as occurs in transmission electron microscopy studies. This increased stability makes them an intriguing material when considering their formation as alteration products of UO_2_, as they may provide a means to securely isolate radiotoxic cationic species present in SNF in an accident scenario.^29^ As such, there has been a concerted effort to understand the formation mechanisms and structural complexities of these materials. This led to the successful incorporation of a range of lanthanides, as illustrated in Figure 1. From these studies, some key insights have been made, most notable of which is the correlation between the ionic radius of the lanthanide species and the resulting structures or space groups adopted by the UOHF material. In incorporating Er and Lu (*R*_CN=8_ ≤ 1.004 Å),^26,28^ UOHF materials have been found to adopt a low symmetry triclinic *P*-1 space group. On the other hand, reported UOHF-Dy/Gd/Eu/Sm materials (*R*_CN=8_ ≥ 1.027 Å) have revealed the frameworks adopt higher symmetry structures, either the monoclinic (*C*2) or orthorhombic (*C*222_1_) space groups.^26,27,31^

**Figure 1:**
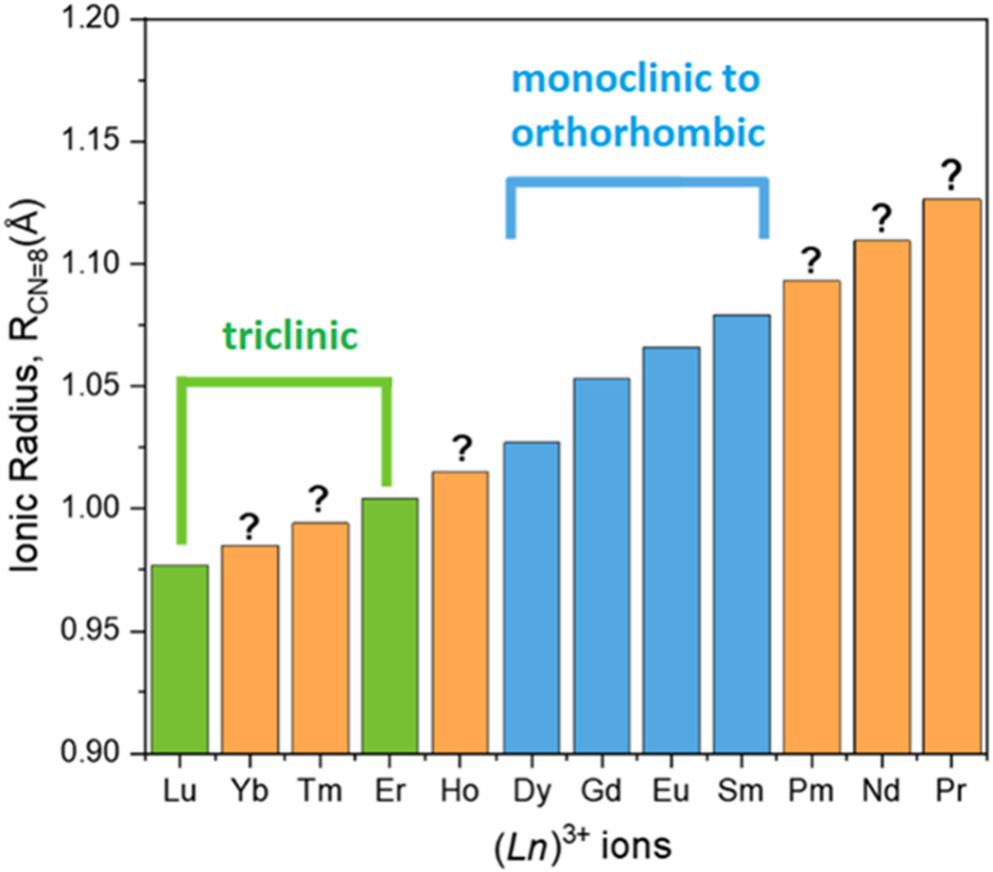
Correlation of ionic radius and UOHF-Ln structures: low symmetry (triclinic) in green, high symmetry (monoclinic to orthorhombic) in blue, and currently unknown in orange.

Two questions remain when examining the reported structures: at exactly which point do these UOHFs switch from adopting the high symmetry structures to the low symmetry structures (*R*_CN=8_ 1.004 Å ≤ *x* ≤ 1.027 Å), and whether there is an upper bound for which lanthanides can successfully be incorporated into these framework-type materials. To best answer the latter question, given the scarcity of promethium, the successful synthesis and structural characterization of Pr- and Nd-containing UOHF materials is vital. Herein, we report the syntheses and characterization of two new UOHF compounds successfully incorporating Pr^3+^ and Nd^3+^ ions (**UOHF-Pr** and **UOHF-Nd**). Synchrotron single-crystal X-ray diffraction analysis revealed that both materials crystallized in the orthorhombic *C*222_1_ space group, with the frameworks constructed from β-U_3_O_8_-type layers bridged by uranium polyhedra, with the lanthanide ions found within the framework channels. The vibrational modes and uranium valences were also investigated using Raman, diffuse reflectance, and medium-energy X-ray absorption spectroscopies.

## 2 Experimental Section

### 2.1 Synthesis of Materials

Uranyl nitrate hexahydrate containing natural isotopic ratios of uranium was used for the synthesis of compounds **UOHF-Pr** and **UOHF-Nd**. Compounds with uranium are radioactive and should be handled in appropriately regulated laboratories. All other chemicals of A.R. grade were sourced from Sigma-Aldrich and used as received.

#### 2.1.1 Pr_1.5_(H_2_O)_6_[(UO_2_)_10_UO_13_(OH)_4_] (UOHF-Pr)

Praseodymium nitrate hexahydrate, Pr­(NO_3_)_3_·6H_2_O (0.143 g, 0.33 mmol), and uranyl nitrate hexahydrate (0.0828 g, 0.165 mmol) were dissolved in 5 mL of deionized water, and the solution pH was adjusted to 4.07 by the dropwise addition of 0.01 M NaOH. The solution was transferred to a 45 mL Teflon vessel, sealed in a steel autoclave, and heated to 240 °C for 72 h. The solution was then cooled, at a rate of 5 °C/h, to room temperature. Vibrant orange needle-like crystals of **UOHF-Pr** were obtained that were collected by filtration, washed with deionized water, and dried at ambient temperature, with 78 wt % (0.041 g) yield. The pH of the final solution was 3.70.

#### 2.1.2 Nd_1.5_(H_2_O)_6_[(UO_2_)_10_UO_13_(OH)_4_] (UOHF-Nd)

Neodymium nitrate hexahydrate, Nd­(NO_3_)_3_·6H_2_O (0.145 g, 0.33 mmol), and uranyl nitrate hexahydrate (0.0828 g, 0.165 mmol) were dissolved in 5 mL of deionized water, and the solution pH was adjusted to 4.48 by the dropwise addition of 0.01 M NaOH. The solution was transferred to a 45 mL Teflon vessel, sealed in a steel autoclave, and heated to 240 °C for 72 h. Upon completion, the solution was cooled to room temperature at a rate of 5 °C/h. Vibrant orange needle-like crystals of **UOHF-Nd** were obtained by filtration, with a final pH of the solution being 3.66. The isolated crystals were washed with deionized water and dried at ambient temperature, with 82 wt % (0.043 g) yield.

### 2.2 Characterization

#### 2.2.1 Synchrotron Single-Crystal X-ray Diffraction (SC-XRD)

Suitable single crystals were harvested manually, and the single-crystal X-ray diffraction data for **UOHF-Pr** (CCDC-2432943) and **UOHF-Nd** (CCDC-2432944) were collected at 100(2) K on the MX2 beamline^32^ at the Australia Synchrotron. A silicon double crystal monochromator was utilized to give X-rays with λ = 0.71079 Å. Data integration and reduction were undertaken with XDS.^33^ Absorption corrections were applied to the data using the SADABS program.^34^ The structures were solved by direct methods^35^ and refined with SHELXL-2014 ^36^ using the Olex^2^ graphical user interface.^37^ All, but the hydrogen atoms were located on the electron density maps and refined anisotropically. The suitable single crystals are in fine needles, making the crystallographic study very challenging, even with the synchrotron X-ray source. Consequently, only one-circle data collections were possible with less data redundancy for effective absorption corrections, which is evidenced by the presence of large ripples around the uranium atoms.

#### 2.2.2 Scanning Electron Microscopy (SEM)

The crystal morphologies and elemental compositions were analyzed by using SEM coupled with energy dispersive spectrometry (EDS). Samples were carbon-coated and examined on a TESCAN FERA3 SEM instrument, operating at an accelerating voltage of 15 keV, with an attached ThermoFisher UltraDry EDS X-ray microanalysis system. EDS point analyses were carried out on relatively flat crystal surfaces with a Cu standard for calibration.

#### 2.2.3 Powder X-ray Diffraction (PXRD)

Powder X-ray diffraction (PXRD) data were collected on a Bruker D8 Focus diffractometer equipped with Cu Kα (λ = 1.5418 Å) radiation, in the range 5° < 2θ < 60°, with a step size of 0.02° (2θ) and an acquisition time of 2 s per step. A LeBail fit of the collected powder diffraction data was fit to the orthorhombic cell found in the SC-XRD study using Topas.^38^

#### 2.2.4 U M_4_-Edge Medium-Energy X-ray Absorption Spectroscopy (XAS)

X-ray absorption data were collected at the U M_4_ edge on the medium-energy X-ray (MEX-1) beamline at the Australian Synchrotron. Samples were diluted to 1000 ppm in cellulose and pressed into pellets (6 mm × 3 mm × 1 mm thickness) contained within nonadhesive Kapton film (8 μm thickness). Analyses were carried out in fluorescence mode at room temperature. Indium metal reference foil was used for energy calibration (3730 eV). Background normalization and subtraction, and data smoothing were achieved using Athena^39^ and Origin.

#### 2.2.5 Diffuse Reflectance Spectroscopy (DRS)

Absorption spectra in both the UV–visible and near-infrared (NIR) regions were recorded on an Agilent Cary 5000 spectrophotometer equipped with a Labsphere Biconical Accessory and referenced to a Labsphere certified standard.

#### 2.2.6 Raman Spectroscopy

Raman spectra were collected on a Renishaw inVia spectrometer equipped with a 785 nm excitation Ar laser in the range 2000–100 cm^–1^ with a spectral resolution of ∼1.7 cm^–1^.

## 3 Results and Discussion

### 3.1 Materials Synthesis and Microstructure

Compounds **UOHF-Pr** and **UOHF-Nd** were successfully synthesized via a hydrothermal reaction of uranyl nitrate with the corresponding lanthanide nitrate in a 1:2 molar ratio. Adjusting the starting solution pH with a freshly prepared dilute NaOH solution to 4.0–4.5, which resulted in final solution pHs of 3.66 to 3.70, was found to be optimal for the growth of the UOHF phases.

Analysis of the isolated crystals by SEM (Figure 2a,2b) revealed needle crystal morphologies for both **UOHF-Pr** and **UOHF-Nd**. The U:Ln atomic ratios were determined via the EDS (Figure 2), with a U:Pr/Nd atomic ratio of 7–8 revealed. This ratio is strongly indicative of the formation of a UOHF structure.^26–28^ The SC-XRD results, described below, show the Ln:U ratio to be 1:7.33.

**Figure 2:**
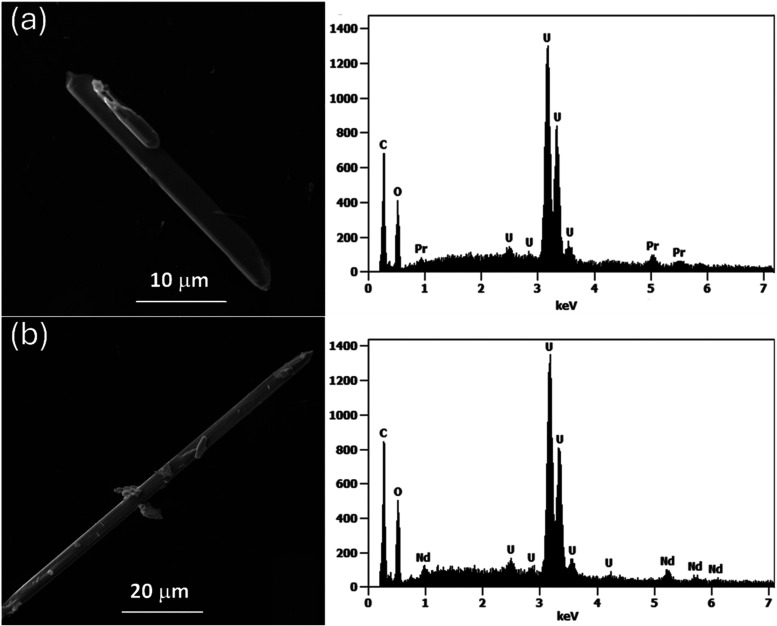
Backscattered SEM images of **UOHF-Pr** (a) and **UOHF-Nd** (b), revealing their needle crystal morphologies. Their corresponding EDS spectra are included on the right, confirming the presence of U and Pr/Nd with U:Pr/Nd atomic ratio of ∼7.5.

The PXRD data were well fit to the orthorhombic cell calculated from the SC-XRD data with a LeBail fit (Figures S1–S2, SI), demonstrating the studied crystals to be representative of the bulk phase.

### 3.2 Crystal Structures

The single-crystal data and structure refinement for the two materials are described in Table 1, with selected bond lengths and angles provided in Tables S1 and S2 (SI) for **UOHF-Pr** and **UOHF-Nd**, respectively. Both **UOHF-Pr** and **UOHF-Nd** crystallize in the orthorhombic space group *C*222_1_, being isostructural. Of immediate interest was the observed *C*222_1_ space group, with a comparison of these materials to the previously reported **UOHF-Eu**,^27^ **UOHF-Gd**,^27^ and **UOHF-Dy** ^26^ showing a close match, strongly suggesting that the same high symmetry UOHF structure was obtained. The asymmetric units for both structures feature seven distinct uranium centers, five of which (U1, U4, U5, U6, and U7) assume pentagonal bipyramidal coordination polyhedra. The remaining U2 and U3 adopt an octahedral coordination geometry. The lanthanide cations are present with partial occupancies across two sites and take on either a 7-fold distorted pentagonal bipyramidal or 8-fold bicapped trigonal prismatic geometry.

**Table 1:** Structural Refinement Detail for Compounds **UOHF-Pr** and **UOHF-Nd**

compound	**UOHF-Pr**	**UOHF-Nd**
CCDC	2432943	2432944
empirical formula	Pr_1.5_U_11_O_42_	Nd_1.5_U_11_O_42_
formula weight	3501.70	3506.69
crystal system	Orthorhombic	Orthorhombic
space group	*C*222_1_ (#20–1)	*C*222_1_ (#20–1)
*a* (Å)	11.628(2)	11.634(2)
*b* (Å)	21.006(4)	20.994(4)
*c* (Å)	14.271(3)	14.254(3)
volume (Å^3^)	3485.9(12)	3481.5(12)
*Z*/μ(mm^–1^)	4/53.073	4/53.278
min./max. 2θ [deg]	3.878/49.988	3.88/49.994
*d*_calcd_ (g cm^–3^)	6.672	6.690
GOF	1.109	1.053
final *R*_1_ ^a^ [*I* > 2σ(*I*)]	0.0481	0.0550
final w*R*_2_ ^b^ [*I* > 2σ(*I*)]	0.1237	0.1394

a *R*_1_ = ∑∥*F*_o_| – |*F*_c_∥/|*F*_o_|.

b w *R*_2_ = ∑[w­(*F*_0_^2^ – *F*_c_^2^)^2^]/∑[w­(*F*_0_^2^)^2^]^1/2^.

The framework backbone is composed of sheets of U-centered octahedra and pentagonal bipyramidal coordination polyhedra. The sheets have a β-U_3_O_8_-type topology where the 7-coordinate U centers (U1, U4, U5, and U6) form chains which extend along the *b*-axis via edge- and corner sharing, with these chains separated by the two octahedral U centers (U2 and U3). Double U7 polyhedra link these layers, making up the walls of the 1D channels which extend along the *a*-axis and giving rise to the framework-type structure (Figure 3).

**Figure 3:**
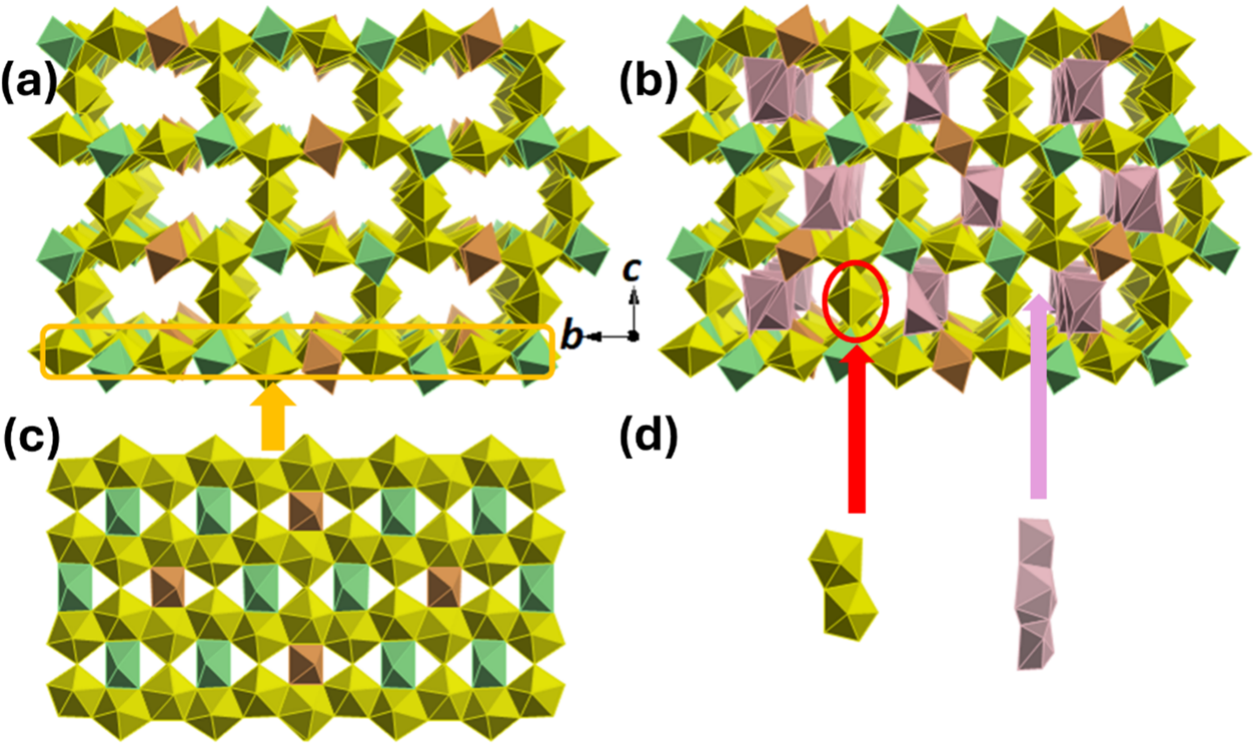
Representation of the crystal structures of **UOHF-Pr** and **UOHF-Nd**: polyhedral views of the frameworks viewed down the *a*-axis (a), backbone structure and Pr/Nd present in the channels (b), a β-U_3_O_8_ type layer with the octahedral U2 (orange) and U3 (green) (c), and the double U polyhedra linking the layers, and the disordered Pr/Nd polyhedral (d).

The U2 and U3 centers both adopt an octahedral coordination geometry but exhibit stark differences. The U3 center has the expected uranyl characteristics, with two short bond lengths (1.84(3) and 1.90(2) Å for **UOHF-Pr**, 1.84(2) and 1.86(2) Å for **UOHF-Nd**) and the corresponding trans-uranyl bond angles (O=U=O) of 175.6(10) and 175.8(10)°, respectively. This contrasts with the U2 octahedra that lacks a uranyl bond, rather, it contain three pairs of U–O bonds with respective bond lengths of 1.99(2), 2.02(2), and 2.21(2) Å for **UOHF-Pr**, and 1.99(2), 2.01(2), and 2.26(2) Å for **UOHF-Nd**. Such uranium centers, defined as having a tetraoxide core, have been reported in the other high symmetry UOHF materials.^26,27^ Interestingly, in these other high symmetry structures, this 6-coordinate U center has been identified as existing as partially U^5+^. As such, bond valence sum (BVS) calculations for the uranium centers were performed assuming the presence of U^6+^ (*R*_U–O_ = 2.051; *B* = 0.519).^9,40^ BVS calculations for the cations and anions are presented in Tables S3 and S4 (SI). The 7-coordinate U centers (U1, U4, U5, U6, U7) were consistent across both **UOHF-Pr** and **UOHF-Nd**, and confirmed their existence as U^6+^ [U1 (5.96), U4 (6.00), U5 (6.09), U6 (5.95) and U7 (6.01) for **UOHF-Pr**; U1 (5.95), U4 (6.07), U5 (6.06), U6 (6.03) and U7 (5.97) for **UOHF-Nd**]. As with the previously reported structures, the BVS of both U2 and U3 were calculated to be slightly less than the expected value of 6. The U2 octahedral coordination sites for **UOHF-Pr** and **UOHF-Nd** are calculated to have BVS values of 5.84 and 5.75, respectively, while the U3 centers gave BVS values of 5.81 and 5.80. While not conclusive, given the presence of U^5+^ in the analogous UOHF materials, this was highlighted as warranting further study. Most of the oxygens in the structure were confirmed to exist as oxo (O) species, with two OH sites (O9 and O22 for **UOHF-Pr**; O7 and O13 for **UOHF-Nd**) and three H_2_O ligands, coordinated solely to the Pr and Nd centers (O18, O19 and O21 for **UOHF-Pr**; and O20, O21 and O22 for **UOHF-Nd**). As such, the two materials can be formulated as Pr_1.5_(H_2_O)_6_[(UO_2_)_10_UO_13_(OH)_4_] (*Z* = 4) for **UOHF-Pr** and Nd_1.5_(H_2_O)_6_[(UO_2_)_10_UO_13_(OH)_4_] (*Z* = 4) for **UOHF-Nd**.

Crystallizing in the orthorhombic *C*222_1_ space group for both **UOHF-Pr** and **UOHF-Nd** confirms the high symmetry lattice trend with the Ln ionic radius (Table 2). Remarkably, the unit cell parameters and cell volumes of these high symmetry structures do not appear to change significantly, even with the incorporation of the much larger Pr^3+^/Nd^3+^ cations. The most notable change is in the total incorporation and occupancies of the *Ln* species, with two half-occupancies of Nd/Pr species observed in this work. Given the increase in ionic radius, it appears that less Ln is required to stabilize the framework, which is reflected in the change in the Ln:U ratio from 1:5.5 to 1:7.3. The change of occupancies to two half-occupancy Ln sites is likely due to the increase in ionic radius, with the Pr^3+^ and Nd^3+^ being able to better span the entirety of the framework channels and not preferentially adopt a position wherein the two uranyl polyhedral layers are slightly closer together. The exception to this trend is the **UOHF-Dy** compound. This may be attributed to its much smaller cation size and appears in proximity to the point wherein the UOHF materials switch from adopting the high symmetry to the low symmetry structures. The successful incorporation of Ho^3+^ ions into a UOHF material would help to elucidate the nature of **UOHF-Dy**.

**Table 2:** Summary of the Key Structural Features for the Characterized UOHF-*Ln* Materials

compound	space group, formula, and cell parameters	asymmetric unit^a^	Ln^3+^ site; *R*_CN=8_ (Å)	refs
UOHF-Pr	*C*222_1_: orthorhombic; Pr_1.5_(H_2_O)_6_[(UO_2_)_10_UO_13_(OH)_4_]	4 U (1), 3 U (1/2)	Pr disordered on 2 sites; 1.126	this work
	*a* = 11.628(2) *b* = 21.006(4) *c* = 14.271(3)	2 Pr (0.5)		
UOHF-Nd	*C*222_1_: orthorhombic; Nd_1.5_(H_2_O)_6_[(UO_2_)_10_UO_13_(OH)_4_]	4 U (1), 3 U (1/2)	Nd disordered on 2 sites; 1.109	this work
	*a* = 11.634(2), *b* = 20.994(4), *c* = 14.254(3)	2 Nd (0.5)		
UOHF-Sm	*C*2: monoclinic; Sm_2_(H_2_O)_7_[(UO_2_)_10_UO_14_(OH)_3_]	11 U (1)	Sm disordered on 2 sites; 1.079	^31^
	*a* = 11.626(2), *b* = 20.975(4), *c* = 14.199(3) Å; β = 90.04(3)^o^	2 Sm (0.65, 0.35)		
UOHF-Eu	*C*222_1_: orthorhombic; Eu_2_(H_2_O)_7_[(UO_2_)_10_UO_14_(OH)_3_]	4 U (1), 3 U (1/2)	Eu disordered on 2 sites; 1.066	^27^
	*a* = 11.629(2), *b* = 20.973(4), *c* = 14.170(3) Å	Eu (0.65, 0.35)		
UOHF-Gd	*C*222_1_: orthorhombic; Gd_2_(H_2_O)_7_[(UO_2_)_10_UO_14_(OH)_3_]	4 U (1), 3 U (1/2)	Gd disordered on 2 sites; 1.053	^27^
	*a* = 11.624(2), *b* = 20.972(4), *c* = 14.181(3) Å	Eu (0.65, 0.35)		
UOHF-Dy	*C*222_1_: orthorhombic; Dy_1.36_(H_2_O)_6_[(UO_2_)_10_UO_13_(OH)_4_]	4 U (1), 3 U (1/2)	Dy disordered on 2 sites; 1.022	^26^
	*a* = 11.625(2), *b* = 20.980(4), *c* = 14.182(3) Å	Dy (0.44, 0.24)		
UOHF-Er	*P*-1: triclinic; Er_2_(H_2_O)_8_[(UO_2_)_10_UO_14_(OH)_3_]	5 U (1), 1 U (1/2)	Er on 1 site; 1.004	^28^
	*a* = 8.1060(16), *b* = 11.435(2), *c* = 11.582(2) Å; α = 111.33(3)^o^, β = 102.97(3)^o^, γ = 106.48(3)^o^	Er (1)		
UOHF-Lu	*P*-1: triclinic; Lu_2_(H_2_O)_8_[(UO_2_)_10_UO_14_(OH)_3_]	5 U (1), 1 U (1/2)	Lu on 1 site; 0.977	^26^
	*a* = 8.0460(16), *b* = 11.358(2), *c* = 11.524(2) Å; α = 111.41(3)^o^, β = 103.24(3)^o^, γ = 105.81(3)^o^	Lu (1)		

a U­(1/2): in full occupancy with a center of symmetry.

Importantly, the successful incorporation of Pr^3+^ or Nd^3+^ extends the higher symmetry lattice trend well beyond the previously reported **UOHF-Sm** structure, demonstrating the flexible nature of this high symmetry framework structure.

### 3.3 Electronic Structures and Uranium Valences

Given the uncertainty of the BVS calculations for the octahedral U centers in both materials, the U valence states were explored further using a combination of DRS and XAS. The U­(IV) and U­(V) ions exhibit observable *f–f* transitions in the UV and NIR regions in DRS, arising from the 5*f* ^2^ and 5*f* ^1^ electronic configurations, respectively.^41,42^ While the U­(IV) spectra present both sharp (zero-phonon lines) and broad (vibronic) absorption bands, the U­(V) ion has sharp absorption bands confined to the near-infrared range 1538–833 nm (6500–12000 cm^–1^). Conversely, the U­(VI) ion with 5*f* ^0^ electronic configuration only exhibits strong and broad charge-transfer bands located in the UV–vis and far UV regions.^42–46^

The DRS spectra are depicted in Figure 4, and they reveal broad absorption bands in the UV region, consistent with the majority of uranium centers present as U^6+^ in the framework structures. The NIR region revealed broad peaks in the spectra for both **UOHF-Pr** and **UOHF-Nd** arising from the ^3^F_4_ and ^3^F_3_, and ^4^I_15/2_ transitions from the Pr^3+^ and Nd^3+^ species, respectively.^47,48^ However, evident in both materials is a small, sharp peak at ∼1440 nm. This peak is consistent with that observed for the 6-coordinate nonuranyl U­(V) center, as reported recently,^49^ demonstrating the framework has sufficient structural flexibility to accommodate lower valence U­(V), which appears to be confirmed in both materials.

**Figure 4:**
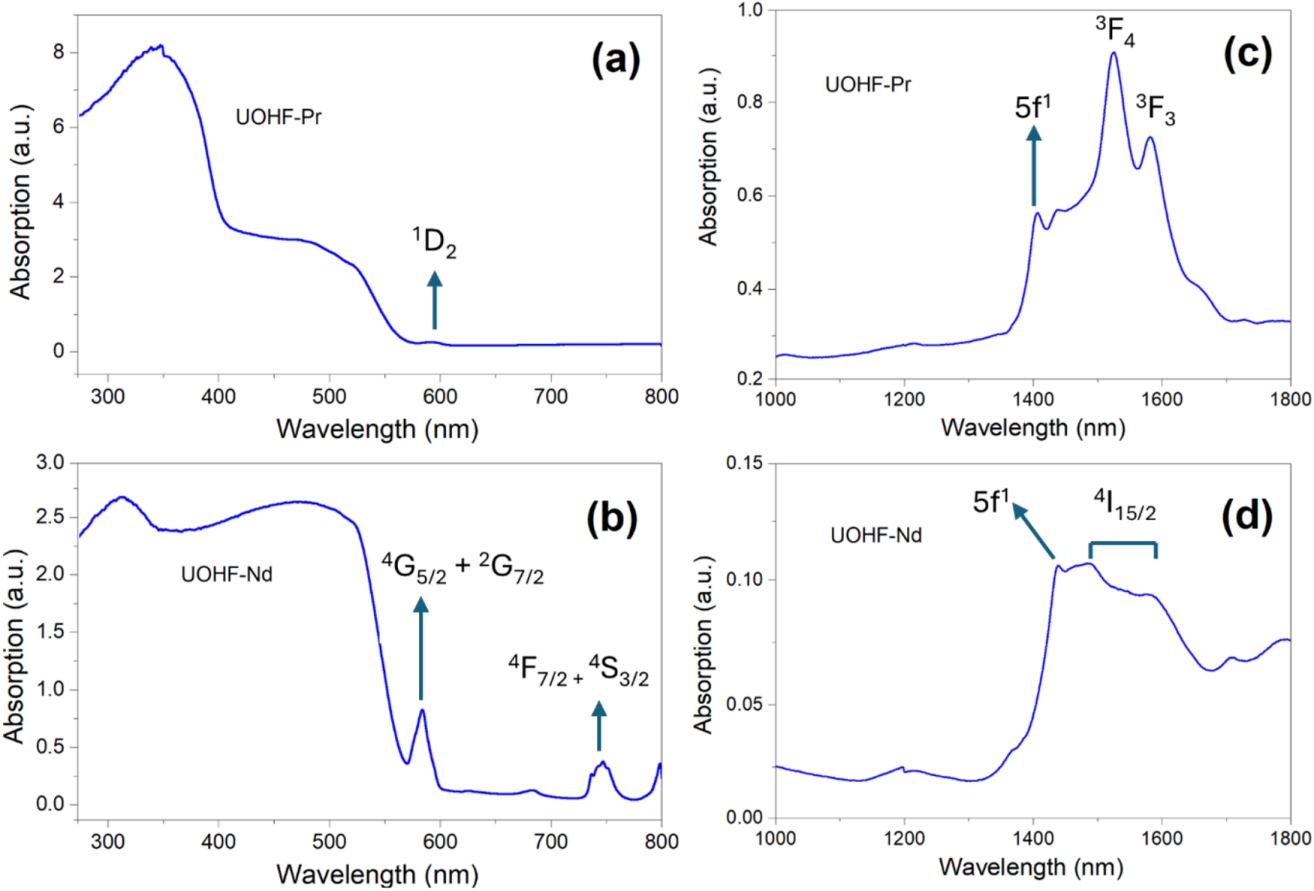
DR spectra of **UOHF-Pr** [(a) and (c)] and **UOHF-Nd** [(b) and (d)] in the UV–vis and the near-infrared regions, respectively. Labeled are the U­(V) 5f^1^ transition, along with the absorption peaks arising from the additional Pr^3+^ and Nd^3+^ f–f transitions.

To further elucidate the presence of U­(V) in these structures, XAS was performed on the **UOHF-Nd** compound on the MEX-1 Beamline at the Australian Synchrotron. The data collection for **UOHF-Pr** was unsuccessful due to sample availability. X-ray Absorption Near Edge Spectroscopy was conducted at the U M_4_-edge (3728 eV), as the M-edge directly probes the unoccupied 5f electronic states and gives sharper spectral features that shift measurably with oxidation state than the higher energy L-edge. Such measurements have proven particularly suitable for differentiating U­(V) from both U­(IV) and U­(VI).^50,51^ The absorption spectra of **UOHF-Nd**, together with U^IV^O_2_, HoU^V^O_4_, BaSr_2_U^VI^O_6_ and U_3_O_8_ as U valent standards, were collected. Figure 5a depicts the U valence standards with expected U oxidation states and shifted absorption maxima from U^4+^ at 3725.7 eV to U^5+^ at 3726.7 eV and U^6+^ at 3727.5 eV, while Figure 5b presents the XAS spectrum for **UOHF-Nd** along with U_3_O_8_, a known U­(V)/U­(VI) mixed valence system. A notable shift from the U^6+^ peak is observed in the **UOHF-Nd** spectrum, with an almost identical spectrum to that of U_3_O_8_. Together with the DRS analysis, it appears to be highly likely that U^5+^ is present in both structures. Unfortunately, HERFD was not accessible for this work. As such, the obtained XANES results were not in high resolution as anticipated.

**Figure 5:**
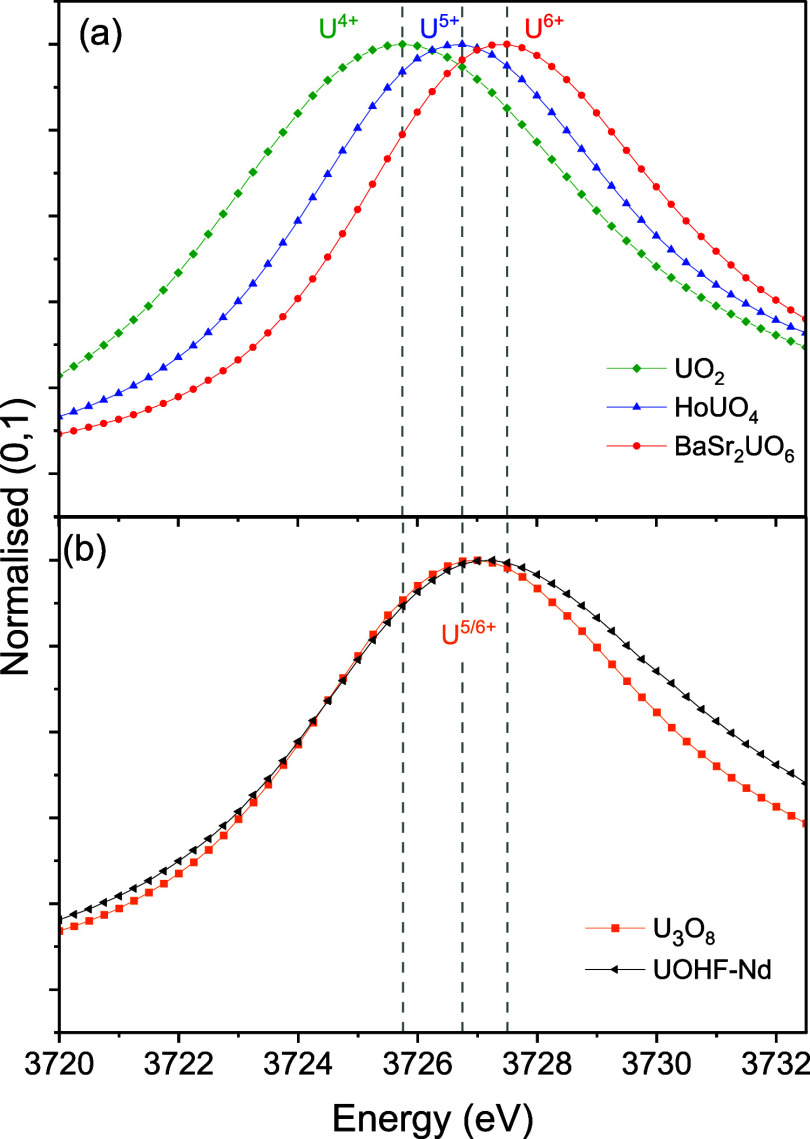
U M_4_ edge spectra for UO_2_ as U^4+^ (green), HoUO_4_ as U^5+^ (blue), BaSr_2_UO_6_ as U^6+^ (red) standards (a), and U_3_O_8_ (orange) as a U^5+^/^6+^ reference and **UOHF-Nd** (black) (b) in the energy range of 3720 to 3732 eV.

### 3.4 Vibrational Modes

Raman spectroscopy was employed to examine the vibrational modes within **UOHF-Pr**, with **UOHF-Nd** being unsuitable due to the strong fluorescence. Distinct vibrational features can be expected for the distinct U centers in the framework, as confirmed by previous studies of UOHF materials in the literature. Notably, the ν_1_(UO_2_)^2+^ should be significant, appearing between 700 and 900 cm^–1^ as strong, sharp peaks.^52^

Similar to an earlier study of **UOHF-Dy**,^26^ the Raman spectrum of **UOHF-Pr** (Figure 6) exhibits four distinct peaks between 704 and 875 cm^–1^, correlating with the ν_1_(UO_2_)^2+^ vibrational modes of the uranyl U = O bonds, reflecting the presence of multiple uranyl centers. Additional broad peaks at 294–524 cm^–1^ arise from γ­[U_3_(OH)_3_] bending vibrations, ν­(U_3_O) bridge elongations, and ν­(U–O_ligand_) vibrations, with lattice vibrations below 200 cm^–1^. The results are in agreement with those of comparable structures in the literature.^26,27^

**Figure 6:**
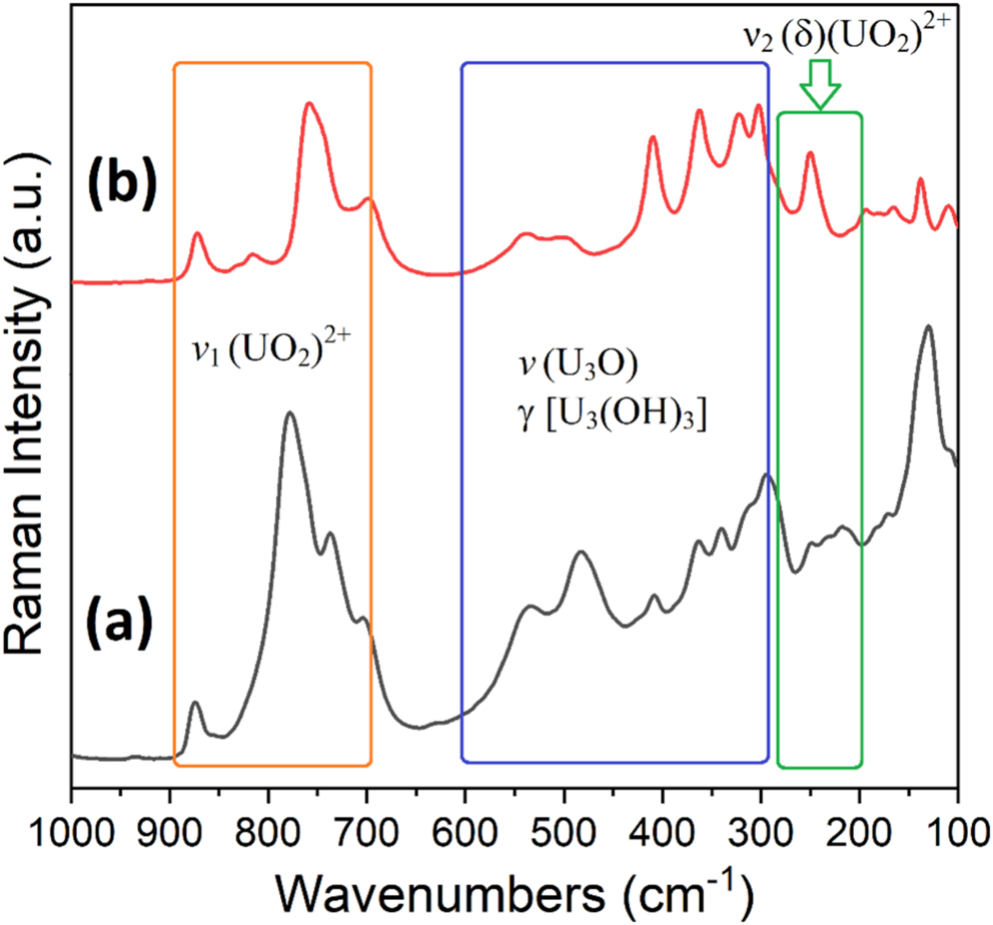
Raman spectra of **UOHF-Pr** (a) and an earlier reported **UOHF-Dy** ^26^ (b) over the region 1000–100 cm^–1^.

### 3.5 Implications and Perspectives

The two compounds synthesized and reported in this work add to a continuing body of research investigating the stability of Ln^3+^ ions incorporated into UOH frameworks.^26–28,31^ The successful inclusion of the Nd^3+^ and Pr^3+^ cations, with relatively larger ionic radii, into the orthorhombic *C*222_1_ structure adopted by these UOHF materials is further evidence that the ionic radius of the Ln^3+^ cations is a dominant driving force influencing the formation and stabilization of these UOHF structures. By filling the gaps for early lanthanide ions illustrated in Figure 1, there is a clear delineation between the high and low symmetry structures, with further work exploring the uncertainty of Ho^3+^ required to confirm the exact point at which this change in symmetry occurs.

The incorporation of the larger Ln^3+^ cations highlights the flexibility of the β-U_3_O_8_ type layer, which can undergo minor topological changes to accommodate the change in ionic radius, as illustrated in Figure 7. The most notable difference between the two structures is the manner in which the Ln^3+^ cations are incorporated, with the larger Ln^3+^ lying along the center of the channels and disordered about two sites, whereas the smaller Ln^3+^ species preferentially adopt a position closer to the channel walls and are present in full occupancies at each position. To further explore the upper tolerance of these systems, studies involving the largest of the lanthanide series, Ce and La, are still required.

**Figure 7:**
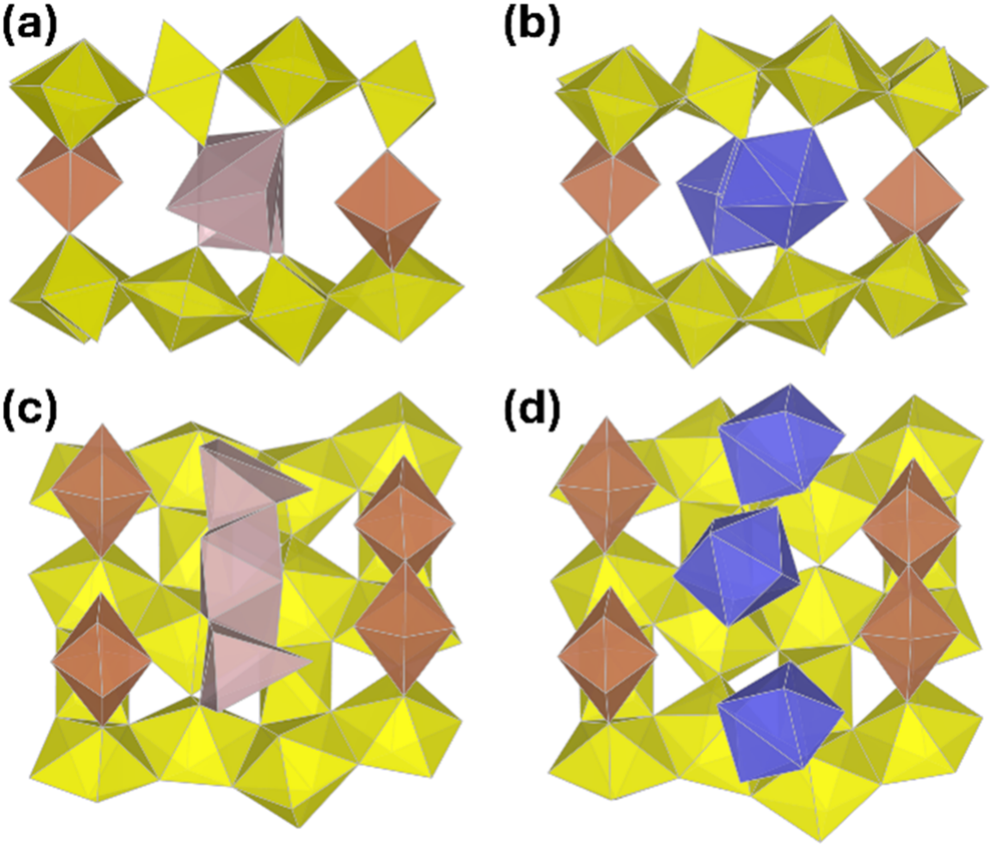
Representation of the crystal structures of **UOHF-Pr** (in the high symmetry *C*222_1_ space group) and **UOHF-Er** (in the low symmetry P-1 space group): polyhedral views of the framework channels for **UOHF-Pr** (a) and **UOHF-Er** (b), and perspective views along the framework channels highlighting the differing coordination of the lanthanide species within the respective structures (c) for **UOHF-Pr** and (d) for **UOHF-Er**. The β-U_3_O_8_ type layers are in yellow, the double U polyhedra linking the layers are in brown, Pr is in purple, and Er is in blue.

The broader implications arising from the confirmation that the ionic radius of the lanthanide cation plays a critical role in directing the alteration chemistry of UO_2_ are toward the capacity for these materials to encapsulate radiotoxic minor actinides such as Am^3+^ and Cm^3+^. While not present in large abundance in SNF, Am^3+^ is a significant factor when considering the decay chain of ^241^Pu, which presents a more significant challenge when considering long-term storage in geological repositories.^53^ Several studies have reported the use of Eu^3+^ and Nd^3+^ as surrogates to Am^3+^,^29,54^ given the similarities in valence and ionic radii (Eu^3+^ *R*_CN=8_ = 1.066 Å; Nd^3+^ *R*_CN=8_ = 1.109 Å; Am^3+^ *R*_CN=8_ = 1.09 Å).^55^ In addition to Gd^3+^ which is typically considered as a lanthanide analogue to Cm^3+^,^53^ Nd^3+^ is more commonly used to model Cm^3+^.^56^ Thus, we can postulate that the **UOHF-Nd** structure reported could incorporate Am^3+^/Cm^3+^ in much the same way, into this stable framework-type structure. Given the hazard these radiotoxic minor actinides pose to the environment and human health, studies into their possible encapsulation into such a material are warranted.

## 4 Conclusions

Two new uranium oxide hydrate frameworks, containing either Pr^3+^ or Nd^3+^ ions, have been successfully synthesized under hydrothermal conditions at 240 °C using uranyl nitrates and the corresponding lanthanide nitrates. Their crystal structures were solved using synchrotron SC-XRD and a combination of Raman spectroscopy, DRS, XAS, and SEM-EDS to probe their microstructure and spectroscopic properties. The two materials, **UOHF-Pr** and **UOHF-Nd**, crystallize in the orthorhombic *C*222_1_ space group, following trends observed in other members of the UOHF-*Ln* family. The framework structure is constructed by β-U_3_O_8_-type sheets, bridged by double pentagonal bipyramidal U polyhedra to form 1D channels, within which lie the Pr^3+^/Nd^3+^ ions. BVS calculations suggested the possible presence of U­(V) in both structures, which was confirmed by a combination of DRS and U M_4_ XAS.

The hypothesized trend that lanthanide species with ionic radii greater than 1.027 Å drive the formation of a high symmetry structure was upheld in this study, with the crystal structures of both **UOHF-Pr** and **UOHF-Nd** closely resembling those reported for UOHF-Dy/Sm/Eu/Gd. This work clearly highlights that the structure adopted by these UOHF materials, seen as possible alteration products of UO_2_ under oxidation conditions, is driven at least partially by the ionic radius of the cationic species present alongside UO_2_ under these conditions. This has implications for the understanding of U geochemistry and potential alteration pathway of SNF if exposed to the surrounding environment, such as in an accident scenario. Future work is still required to elucidate several unknowns plaguing these UOHF-*Ln* systems, with a need to extend these findings to minor actinide-based systems.

## Supplementary Material







## Data Availability

The data underlying this study are not publicly available due to an unfinished project. The data are available from the corresponding author upon reasonable request.

## Notes

PXRD patterns and tables of bond lengths, angles, and BVSs (PDF)
UOHF-Pr (CIF)
UOHF-Nd (CIF)
