# Supplementary material for: Expanding Uranium Oxide Hydrate Frameworks toward Early Lanthanides: Cases for Pr(III) and Nd(III) Ions
Source: ACS Omega. 2025 Aug 20;10(34):38531–9. doi: 10.1021/acsomega.5c02821 (PMC12409525; doi:10.1021/acsomega.5c02821)
Supplement: Supplementary file 1 [file ao5c02821_si_001.pdf]

Supporting Information

Expanding Uranium Oxide Hydrate Frameworks towards the Early Lanthanides: Cases for Pr(III) and Nd(III) Ions

Maria K. Nicholas,[a,b] Timothy Ablott,[b] Jeremy Wykes,[c] Brendan J. Kennedy[a] and Yingjie Zhang[b]*

[a] *School of Chemistry, The University of Sydney, Camperdown, NSW 2006, Australia.*

[b] *Australian Nuclear Science and Technology Organisation, Locked Bag 2001, Kirrawee DC, NSW 2232, Australia.*

[c] *Australian Synchrotron, ANSTO, 800 Blackburn Road, Clayton, VIC 3168, Australia.*

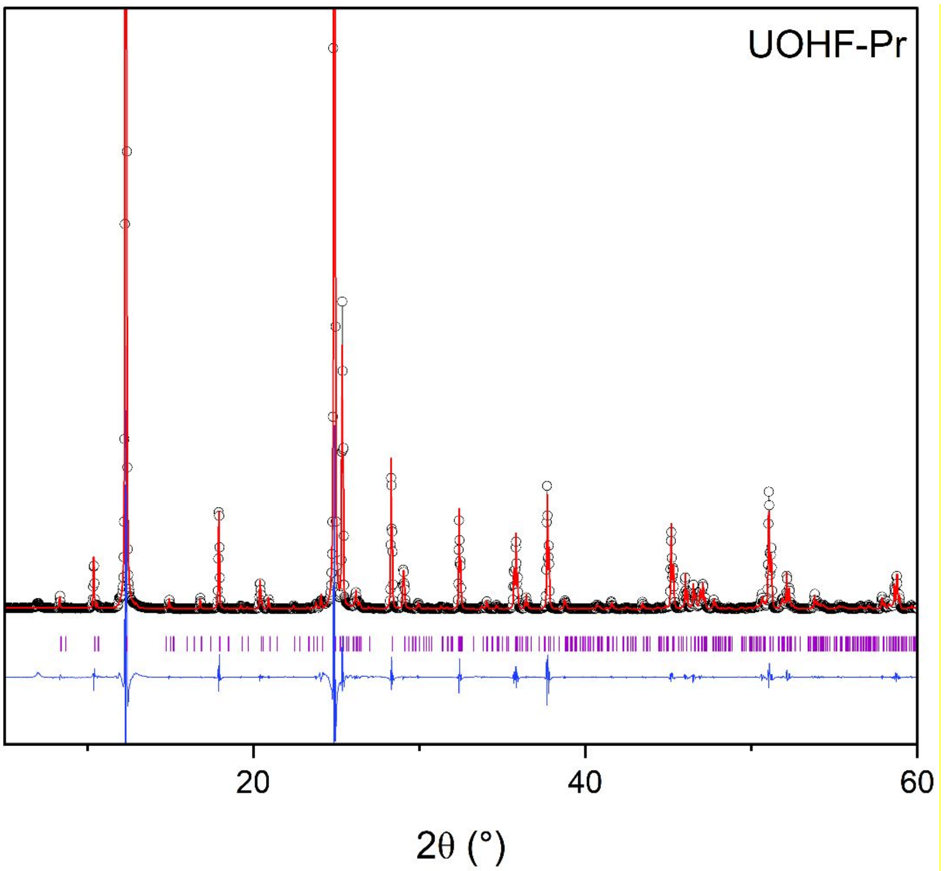

Figure S1. A LeBail fit of the PXRD data for compound **UOHF-Pr**: black circles represent the observed data points, the red lines are the LeBail fit, vertical markers show the peak positions for the corresponding structure, and the blue curve underneath shows the difference between the measured and calculated data.

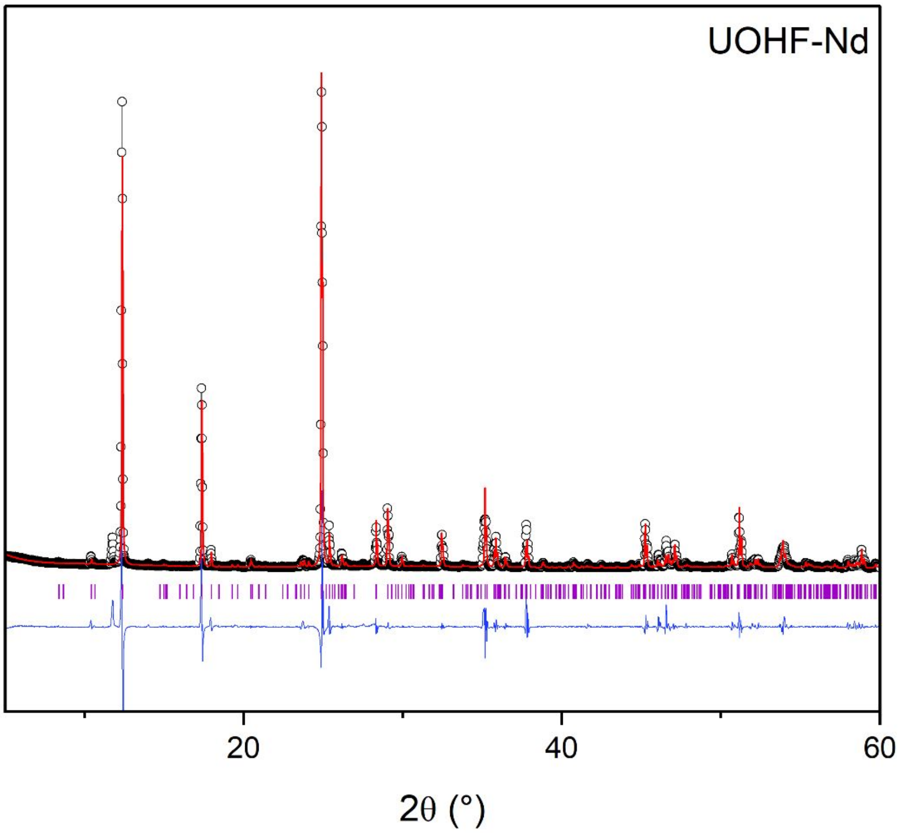

Figure S2. A LeBail fit of the PXRD data for compound **UOHF-Nd**: black circles represent the observed data points, the red lines are the LeBail fit, vertical markers show the peak positions for the corresponding structure, and the blue curve underneath shows the difference between the measured and calculated data.

Table S1. Selected bond lengths and angles for **UOHF-Pr**.

U1-O1	1.82(2)	U2-O5[4]	1.99(3)	U3-O4	2.19(3)	U4-O4	2.28(2)
U1-O2	1.96(3)	U2-O5	1.99(3)	U3-O7	2.19(3)	U4-O4[4]	2.28(2)
U1-O3	2.18(2)	U2-O13[3]	2.02(2)	U3-O8	1.90(2)	U4-O5	2.45(2)
U1-O4	2.31(2)	U2-O13[5]	2.02(2)	U3-O10	2.23(2)	U4-O5[4]	2.45(2)
U1-O5	2.41(2)	U2-O22[3]	2.21(2)	U3-O12[1]	2.21(3)	U4-O6[4]	1.81(3)
U1-O7[1]	2.27(2)	U2-O22[5]	2.21(2)	U3-O20[6]	1.84(3)	U4-O6	1.81(3)
U1-O12[1]	2.26(2)			O20=U=O8	175.6(10)°	U4-O9	2.31(5)
O1=U=O2	175.7(10)°					O6=U=O6	179.8(17)°
U5-O9	2.33(5)	U6-O3[7]	2.27(2)	U7-O2[2]	2.39(2)	Pr1-O11	2.62(2)
U5-O10	2.334(19)	U6-O7	2.31(2)	U7-O2	2.48(3)	Pr1-O14[4]	2.75(3)
U5-O10[4]	2.334(19)	U6-O10	2.33(2)	U7-O3	2.323(19)	Pr1-O18	2.205(15)
U5-O13[4]	2.39(3)	U6-O11	1.81(3)	U7-O8[2]	2.38(2)	Pr1-O19	2.45(2)
U5-O13	2.39(3)	U6-O12	2.27(2)	U7-O15	1.762(19)	Pr1-O20	2.41(2)
U5-O14[4]	1.79(3)	U6-O13	2.42(2)	U7-O16	1.775(18)	Pr1-O21	2.24(3)
U5-O14	1.79(3)	U6-O17[7]	1.89(2)	U7-O17	2.417(19)	Pr1-O22	2.51(3)
O14=U=O14	173.3(16)°	O11=U=O17	175.1(10)°	O15=U=O16	177.7(10)°		
Pr2-O1	2.62(2)	Pr2-O1	2.62(2)	Pr2-O6	2.69(3)	Pr2-O6	2.69(3)
Pr2-O21	2.34(2)	Pr2-O21	2.34(2)	Pr2-O22	2.73(3)	Pr2-O22	2.73(3)

[1]-1/2+X,3/2-Y,1-Z; [2]-X,+Y,1/2-Z; [3]-1+X,+Y,+Z; [4]+X,1-Y,1-Z; [5]-1+X,1-Y,1-Z; [6]1-X,+Y,3/2-Z; [7]1+X,+Y,+Z; [8]1+X,1-Y,1-Z; [9]1-X,1-Y,1/2+Z; [10]2-X,+Y,3/2-Z

Table S2. Select Bond lengths and bond angles for **UOHF-Nd**.

U1- O1	1.81(2)	U2- O5	1.99(2)	U3- O3	2.26(2)	U4- O4[4]	2.28(2)
U1- O2	1.97(2)	U2- O5[4]	1.99(2)	U3- O4	2.20(2)	U4- O4	2.28(2)
U1- O3	2.24(2)	U2- O6[4]	2.01(2)	U3- O8	1.84(2)	U4- O5	2.44(2)
U1- O4	2.31(2)	U2- O6	2.01(2)	U3- O9	1.86(2)	U4- O5[4]	2.44(2)
U1- O5	2.42(2)	U2- O7	2.26(2)	U3- O10	2.19(2)	U4- O12[4]	1.80(3)
U1- O10[2]	2.26(2)	U2- O7[4]	2.26(2)	U3- O11	2.26(2)	U4- O12	1.80(3)
U1- O17[2]	2.22(2)			O8=U=O9	175.8(10)°	U4- O13	2.32(2)
O1=U=O2	176.9(10)°					O12=U=O12	178.0(14)°
U5- O6[7]	2.40(2)	U6- O3	2.23(2)	U7- O2[11]	2.39(2)	Nd1- O7[12]	2.55(3)
U5- O6[8]	2.40(2)	U6- O6[9]	2.43(2)	U7- O2[5]	2.47(2)	Nd1- O8	2.42(2)
U5- O11	2.30(2)	U6- O10[2]	2.32(2)	U7- O9[11]	2.42(2)	Nd1- O14[10]	2.74(3)
U5- O11[4]	2.30(2)	U6- O11[2]	2.34(2)	U7- O16	2.45(2)	Nd1- O15[11]	2.58(2)
U5- O13	2.35(2)	U6- O15	1.80(2)	U7- O17	2.31(2)	Nd1- O20	2.186(13)
U5- O14	1.80(3)	U6- O16	1.89(2)	U7- O18	1.77(2)	Nd1- O21	2.31(2)
U5- O14[4]	1.80(3)	U6- O17	2.25(2)	U7- O19	1.77(2)	Nd1- O22	2.38(2)
O14=U=O14	174.5(14)°	O15=U=O16	175.9(10)°	O18=U=O19	176.7(10)°		
Nd2- O1[12]	2.63(2)	Nd2- O1	2.63(2)	Nd2- O7[12]	2.61(3)	Nd2- O7	2.61(3)
Nd2- O12[13]	2.66(3)	Nd2- O12[4]	2.66(3)	Nd2- O21	2.35(2)	Nd2- O21[12]	2.35(2)

[1]1/2-X,3/2-Y,-1/2+Z; [2]1/2+X,3/2-Y,1-Z; [3]1+X,+Y,+Z; [4]+X,1-Y,1-Z; [5]-1/2+X,3/2-Y,1-Z; [6]-1/2+X,-1/2+Y,+Z; [7]-1+X,1-Y,1-Z; [8]-1+X,+Y,+Z; [9]-1/2+X,1/2+Y,+Z; [10]-X,+Y,3/2-Z; [11]1/2-X,3/2-Y,1/2+Z; [12]1-X,+Y,3/2-Z; [13]1-X,1-Y,1/2+Z

Table S3. Calculated BVS values for cations and anions in **UOHF-Pr**.

	**U1**	**U2**		**U3**	**U4**		**U5**		**U6**	**U7**		**Pr1**	**Pr2**			
**Occ.**	**1**	**1**		**1**	**1**		**1**		**1**	**1**		**0.5**	**0.5**			
**Sym.**	**1**	**1**		**1**	**1**		**2**		**1**	**1**		**1**	**1**			
**CN**	**7**	**6**		**6**	**7**		**7**		**7**	**7**		**7**	**8**		**Σ**	
O1	1.56												0.25	0.25	**1.81**	
O2	1.19									0.44	0.52				**1.63**	
O3	0.78								0.66	0.60					**2.03**	
O4	0.61			0.77	0.64	0.64									**2.02**	
O5	0.50	1.12	1.12		0.46	0.46									**2.09**	
O6					1.59	1.59							0.21	0.21	**1.80**	
O7	0.66			0.77					0.61						**2.03**	
O8				1.34						0.53					**1.87**	
O9					0.61		0.58								**1.19**	**(OH)**
O10				0.71			0.58	0.58	0.58						**1.87**	
O11									1.59			0.25			**1.84**	
O12	0.67			0.74					0.66						**2.06**	
O13		1.06	1.06				0.52	0.52	0.49						**2.07**	
O14							1.65	1.65				0.17			**1.83**	
O15										1.74					**1.74**	
O16										1.70					**1.70**	
O17									1.36	0.49					**1.85**	
O18												0.76			**0.76**	**(H_2O)**
O19												0.39			**0.39**	**(H_2O)**
O20				1.50								0.44			**1.94**	
O21												0.69	0.53	0.53	**1.22**	**(H_2O)**
O22		0.74	0.74									0.33	0.18	0.18	**1.26**	**(OH)**
**Σ**	**5.96**	**5.84**		**5.81**	**6.00**		**6.09**		**5.95**	**6.01**		**3.05**	**2.34**			

Table S4. Calculated BVS values for cations and anions in **UOHF-Nd**.

	U1	U2		U3	U4		U5		U6	U7		Nd1	Nd2			
**Occ.**	**1**	**1**		**1**	**1**		**1**		**1**	**1**		**0.5**	**0.5**			
**Sym.**	**1**	**1**		**1**	**1**		**2**		**1**	**1**		**1**	**1**			
**CN**	**7**	**6**		**6**	**7**		**7**		**7**	**7**		**7**	**8**		**Σ**	
O1	1.59												0.24	0.24	**1.83**	
O2	1.17									0.52	0.45				**1.69**	
O3	0.69			0.67					0.71						**2.07**	
O4	0.61			0.75	0.64	0.64									**2.00**	
O5	0.49	1.12	1.12		0.47	0.47									**2.09**	
O6		1.08	1.08				0.51	0.51	0.48						**2.07**	
O7		0.67	0.67									0.30	0.26	0.26	**1.22**	**(OH)**
O8				1.50								0.44			**1.93**	
O9				1.44						0.50					**1.94**	
O10	0.67			0.77					0.60						**2.03**	
O11				0.67			0.62	0.62	0.57						**1.86**	
O12					1.62	1.62							0.22	0.22	**1.85**	
O13					0.60		0.56								**1.16**	**(OH)**
O14							1.62	1.62				0.18			**1.80**	
O15									1.62			0.28			**1.90**	
O16									1.36	0.46					**1.83**	
O17	0.72								0.68	0.61					**2.01**	
O18										1.72					**1.72**	
O19										1.72					**1.72**	
O20												0.85			**0.80**	**(H_2O)**
O21												0.71	0.52	0.52	**1.09**	**(H_2O)**
O22												0.48			**0.48**	**(H_2O)**
**Σ**	**5.94**	**5.75**		**5.80**	**6.07**		**6.06**		**6.03**	**5.97**		**3.04**	**2.47**			
